# Personal Doses of Cocaine and Coca Paste are Adulterated in Cartagena de Indias (Colombia)

**DOI:** 10.1155/2021/5562315

**Published:** 2021-05-25

**Authors:** Jefferson Urzola-Ortega, Luis Mendoza-Goez, Diofanor Acevedo

**Affiliations:** ^1^Faculty of Medicine, Universidad de Cartagena, Campus Zaragocilla, Cartagena de Indias 130015, Colombia; ^2^Grupo de Investigación en Innovación y Desarrollo Agropecuario y Agroindustrial (IDAA), Universidad de Cartagena, Campus Piedra de Bolívar, Cartagena de Indias 130015, Colombia

## Abstract

Knowledge of drug composition consumed on the streets and the identification and quantification of their adulterants is essential for understanding unexpected side effects, tracking routes, and drug profiling. Therefore, this work aimed to determine the purity and to identify and quantify the main adulterants found in personal doses of cocaine (perico) and coca paste (bazuco) in Cartagena de Indias (Colombia). The data collected in this study describe a first attempt to introduce the qualitative and quantitative analyses of adulterants present in street drugs in Cartagena de Indias to improve surveillance. Through gas chromatography coupled to mass spectrometry (GC-MS), the purity and adulterants were quantified in 45 personal doses of cocaine powder and coca paste. 100% of the personal doses in the city were adulterated; caffeine, phenacetin, and levamisole were the main adulterants identified in cocaine. Besides the above, lidocaine was also found in coca paste. The purity of cocaine varied from 8% to almost 70%, with caffeine ranging from 6% to 42%. In the case of coca paste, the maximum content of cocaine found was 60%, while some samples contained as little as 14%. The results are consistent with other research in terms of the widespread use of caffeine as an adulterant, but they also follow the growing trend of the use of levamisole and phenacetin. The wide range of cocaine content in samples sold in the illicit market could cause undesirable effects on cocaine users who do not know the exact intended dose for consumption; so, this study intends to make these results available not only to academic, public health, and national security agencies but also to tourists entering Cartagena de Indias, so that they are aware of what they are consuming and the risks to which they are exposed.

## 1. Introduction

Drug production and consumption are not problems that involve only developing countries, as today it is considered a global problem [1, 2]. According to the United Nations Office on Drugs and Crime (UNODC) in its World Drug Report 2020, drug use worldwide has been increasing, both in terms of overall numbers and the proportion of the world's population that consumes drugs. According to the report, by 2019, one in 25 people aged 15–64 had used drugs at least once in their lifetime, with 167,000 deaths associated with drug use in 2017 [3].

According to UNODC [3] itself, the effect of the Covid-10 pandemic on drug markets is unknown and difficult to predict, but it could be far-reaching. In its latest World Drug Report in 2020, this United Nations office claimed that the greatest immediate impact on drug trafficking can be expected in countries where large quantities are smuggled on commercial airline flights. Moreover, the UNODC predicted that the increased unemployment and lack of opportunities will make it more likely that poor and disadvantaged people will engage in harmful patterns of drug use, suffer from drug use disorders, and turn to illicit drug-related activities, whether that be production or transportation [3].

Cocaine hydrochloride (cocaine or perico, as it is better known colloquially) is one of the most widely consumed illicit substances worldwide [4, 5]. Cocaine is made from the leaves of the coca plant and is an addictive stimulant that is usually snorted in powder form. It is also consumed orally, intravenously, or by inhalation, depending on whether it is consumed pure or as a residue [6, 7]. Although illegal in most countries, it is still commonly used recreationally worldwide [8, 9].

According to the latest estimates, there were about 23 million cocaine users worldwide, and the highest number is in America. The United States by itself accounts for about half of those users, with 5.5 million users [10]. It is estimated that in 2017, about a quarter of the world's population used cocaine at some point in their lives [3]. This has made the illicit cultivation of the coca plant, which covers some 245,000 ha worldwide, very lucrative, with most of its production in South America, which may have caused a slight increase in the consumption of cocaine and derivatives in recent years in this region [11].

In the case of Latin America, cocaine and its derivatives such as coca paste (cocaine paste, cocaine base, or bazuco, as they are more commonly known) are mostly consumed in Uruguay and Chile. In contrast, Bolivia, Ecuador, and Paraguay report the least consumption [12]. Regarding Colombia, according to the “Colombia Drug Report 2017” published by the Colombian Drug Observatory, the responsibility of the country's Ministry of Justice and Law, there was evidence of a 3.4% increase in consumption of psychoactive substances in general between 2008 and 2013, with marijuana being the most commonly consumed drug, followed by cocaine, coca paste, and ecstasy [13, 14]. It was also found that the consumption of illicit substances is much higher in the interior of the country, with Antioquia, Quindío, and Risaralda being the departments with the highest consumption; while the departments in the Caribbean region such as Bolívar (Department with capital Cartagena de Indias) show a low consumption of illicit substances, but with increasing consumption and relation to the tourist activity of the Tourist and Cultural District of Cartagena de Indias [13].

Coca paste or bazuco has much stronger effects than pure cocaine on its users, who manifest symptoms of anxiety, euphoria, compulsion, and even anorexia during intoxication. The stimulant effect of this substance disappears promptly, causing feelings of anguish, sleepiness, fatigue, and irritability in the consumer. Besides, the coca paste user usually presents preconsumption syndrome, which consists of anxiety, excitement, sweating, tremors, and rectal urgency [15]. In general, the consumption of these psychoactive substances is associated with burns of the face, lips, and hands, dental diseases, bronchitis, sinusitis, and all kinds of pulmonary complications, as well as episodes of violence and criminal behavior caused by addiction, among others [16].

On the other hand, street drugs are typically modified by dilution, contamination, and adulteration. Dilution refers to the addition of inert substances (diluents), contamination to the presence of by-products of the drug manufacturing process (contaminants), and adulteration involves the intentional addition of a pharmacologically active substance without the user being aware of it (adulterants) [17]. Adulterant substances are incorporated into drugs in order to increase the amount of the product, enhance its effect, and in some cases, minimize negative effects of the drug. However, the use of adulterants can cause health risks, as the origin of the adulterants that are added is rarely known [18]. Studies have shown that cocaine is the most adulterated illicit substance, mainly with levamisole [19], phenacetin [20], caffeine [17], and phenacetin [21] as main adulterants to enhance the effect of the drug.

Knowledge of the purity of drugs consumed on the streets and the identification and quantification of their adulterants are essential for understanding overdose, unexpected side effects (toxicity), and potentially fatal reactions. Furthermore, the identification of adulterants provides relevant information for the investigation of trafficking routes and drug profiling [17]. For such reason, the objective of this study was to determine the purity and to identify and quantify the main adulterants found in personal doses of cocaine and coca paste in the city of Cartagena de Indias, one of the most touristic cities worldwide, reason for which, this study intends that the scientific community, state agencies, and the population, in general, can have access to this type of information of relevance to public health. This work shows the first results collected from donated drug samples in order to set the stage for further local mapping opportunities and to obtain an initial snapshot in comparison to what has been reported in other countries.

## 2. Materials and Methods

### 2.1. Materials and Reagents

Cocaine hydrochloride, used as a standard solution in the chemical analysis, was generously donated by the Faculty of Pharmaceutical Sciences of the University of Cartagena. Caffeine, phenacetin, levamisole, and lidocaine were obtained from Sigma-Aldrich (Colombia).

The cocaine and coca paste samples were donated by anonymous consumers between the months of October and November 2018 in the fashion of personal dose as established by the Colombian Government in Law 30 of 1986: “It is a dose for personal use; the amount of marijuana that does not exceed twenty (20) grams; of hashish marijuana that does not exceed five (5) grams; of cocaine or any cocaine-based substance that does not exceed one (1) gram, and of methaqualone that does not exceed two (2) grams.” These were samples that could have been consumed by drug users.

### 2.2. Sample Collection

**Figure 1:**
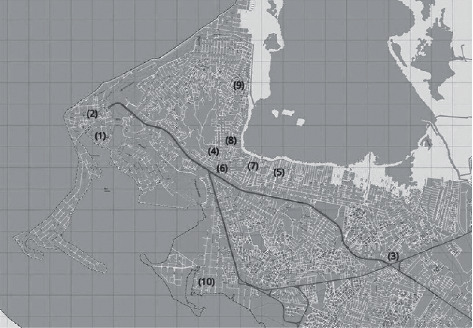
Neighborhoods or “points” from where personal doses were donated in the Tourist and Cultural District of Cartagena de Indias. (1) Getsemaní, (2) Zona Centro, (3) Sector Bomba del Amparo, (4) La Quinta, (5) Líbano, (6) La Esperanza, (7) La Candelaria, (8) La María, (9) San Francisco, and (10) Zapatero.

Personal doses were donated from ten (10) neighborhoods or strategic points in the city of Cartagena de Indias (Colombia). A total of 45 samples were collected. Fifteen (15) personal doses corresponding to cocaine hydrochloride (cocaine or perico) were donated from areas located in the neighborhoods of Getsemaní, Zona Centro, Sector Bomba del Amparo, La Quinta, and Líbano. The remaining thirty (30) personal doses corresponded to coca paste (bazuco) and were donated in the aforementioned areas and five (5) additional areas such as La Esperanza, La Candelaria, La María, San Francisco, and Zapatero neighborhoods.  Figure 1 shows the geographical location of the neighborhoods or “points” from where the personal doses were donated in the city of Cartagena de Indias. According to information provided by the donors themselves, these “points” or areas are known to be the key among regular users of illicit substances in Cartagena de Indias.

### 2.3. Identification and Quantification of Cocaine and Adulterants

To determine the chemical composition of the donated personal doses, solutions of 10 mg of the sample were prepared in 2 mL of ethanol (99.8%, Sigma-Aldrich). An Agilent Technologies 7890A gas chromatograph (Palo Alto, California, USA) coupled to an Agilent Technologies MSD 5975C Inert XL mass selective detector in the split mode (10 : 1) was used. The separation of the analytes was performed on a HP-5MS capillary column of 0.25 mm external diameter, 30 m long, and 0.25 *µ*m internal diameter, with a 5% phenyl-poly (methylsiloxane) apolar stationary phase. The carrier gas used was helium, with a gas velocity of 28.09 cm/s and a volumetric flow rate of 1.2 mL/min. The injector temperature was maintained at 250°C, with the split injection mode (10 : 1) and injection flow rate of 24.2 mL/min. The oven was programmed from 50°C (4 min) to 290°C (2 min), at a rate of 4°C/min. The injector and detector temperatures remained at 250°C and 300°C, respectively. The mass spectra and total ion current (TIC) chromatogram were obtained in a quadrupole, through automatic radiofrequency scanning (full scan). Finally, the m/z mass range was 50 and 350.

For identification purposes, the average mass spectra of each chromatographic peak were compared with the spectra of the analytical standards and with those recorded in the commercial databases (NIST 08 MS Library and Wiley/NBS MS Database). Quantification of the compounds was based on the measurement of the area of the sample peaks, interpolating on the calibration curve.

### 2.4. Data Analysis

Mean and standard deviation were calculated for all data and submitted to analysis of variance (ANOVA). When statistical differences at a significance level of 5% (*p* < 0.05) were found, Fisher's least significant difference (LSD) test was performed to determine the influence of the selected factors. Data processing was carried out using STATGRAPHICS Centurion XVI.I® (Statpoint Technologies, Inc., USA).

## 3. Results and Discussion

It is well known that illicit drugs of abuse are often sold on the street with the main psychoactive ingredient in combination with other substances with the main objective of not only increasing the amount of product but also to enhance its effect, increase addiction, reduce side effects, and even force false-negatives in screening tests [16, 17, 19]. Based on this, this study was carried out with the main objective to chemically analyze a wide range of samples of cocaine hydrochloride (cocaine or perico) and coca paste (bazuco), donated as personal doses by regular users, determining purity and identifying and quantifying adulterants present. Initially, the purity and adulterants found and quantified in samples of cocaine (perico) donated in the neighborhoods of Getsemaní, Zona Centro, Sector Bomba del Amparo, La Quinta, and Líbano in the city of Cartagena de Indias (Figure 1) were discussed. Afterward, the content of cocaine and adulterants found in samples of coca paste (bazuco) in the neighborhoods of Getsemaní, Zona Centro, Sector Bomba del Amparo, La Quinta, Líbano, La Esperanza, La Candelaria, La María, San Francisco, and Zapatero of the city were analyzed (Figure 1).

### 3.1. Purity and Adulterants Found in Cocaine Samples

The purity and adulterants found in cocaine samples from five (5) neighborhoods of Cartagena de Indias are given in Table 1. In Table 1, different superscript letters in the same row indicate statistically significant differences (*p* < 0.05) in the content of the compound in personal doses from different neighborhoods of Cartagena de Indias.

As given in Table 1, 100% of the cocaine samples analyzed from the city of Cartagena de Indias were adulterated; and there were statistically significant differences (*p* < 0.05) in cocaine content in the samples from all the neighborhoods under analysis. Also, the information given in Table 1 indicates that cocaine sold as a powder in the neighborhoods of Getsemaní, Zona Centro, and Sector Bomba del Amparo exhibited the highest cocaine contents, reaching values of 64%, 66%, and 68% of the alkaloid, respectively. On the other hand, in the case of the La Quinta neighborhood, the samples had cocaine concentrations lower than 31%. Finally, the lowest cocaine content detected was found in the samples donated in the Líbano neighborhood, where it did not reach the 10% threshold.

Caffeine was also identified in 80% of the donated samples. Table 1 provides that the samples from the La Quinta and Líbano neighborhoods displayed caffeine concentrations of up to 40%, along with other unidentified components. The Getsemaní and Zona Centro neighborhoods followed with 10% and 6% caffeine, respectively, while in the samples from the Bomba del Amparo sector, this alkaloid could not be detected. The caffeine content was significantly different (*p* < 0.05) in samples from all neighborhoods, except for La Quinta and Líbano (*p* < 0.05).

Caffeine is one of the psychoactive substances most commonly used as an adulterant in illicit drugs, such as cocaine [17]. Previous studies associate the concomitant exposure of cocaine and caffeine to attenuate the excitatory effects of cocaine significantly, thus increasing addiction in those who consume it, aided by the natural addictive effect of caffeine [21, 22]. Another reason for adding caffeine (and the other adulterants found in this study) to cocaine is, as mentioned above, to increase the amount of product and therefore profits [17, 18, 20].

Table 1 provides that the other two adulterants found in the donated cocaine samples were the pharmaceutical drugs phenacetin and levamisole, as in other related studies [20, 21, 23, 24]. Phenacetin was found in 60% of the samples analyzed, with statistically significant differences (*p* < 0.05) of its content in samples from the different neighborhoods where it was detected. According to studies conducted by Brunt et al. [23], the use of this adulterant together with cocaine increases the probability of hallucinations or cardiac effects after consumption. It should be noted that phenacetin is an analgesic and antipyretic with mild euphoric effects. It is a derivative of paracetamol and was withdrawn from the market due to several adverse side effects, such as cardiovascular or renal morbidity and mortality. Due to the above, this drug achieves pain relief that can occur during cocaine use [24].

As for levamisole, it was also present in 60% of the samples analyzed in concentrations ranging from 2% to 15%. The lowest content was detected in samples from Zona Centro, while the highest content was found in samples from Getsemaní. Levamisole is metabolized to aminorex, which is considered an amphetamine-like substance, due to its actions on monoamine transporters. Research provides experimental evidence that levamisole possibly prolongs the effect of cocaine [24, 25]. Other studies described that the use of this adulterant causes anemia, acute renal failure, and neutropenia in those who consume it [18, 23]. According to Karch et al. [26], the first case reports of levamisole-related disease in cocaine users were published in 2010, although levamisole adulteration of cocaine was first recognized several years earlier. These authors also claimed that more than 70% of street cocaine seizures in the United States and the European Union contain levamisole.

In another work, Pichini et al. [27] analyzed by GC-M cocaine samples recently seized by “Carabinieri,” one of the three national police forces from Italy (Carabinieri, State Police, and Financial Police), from Italian regions: Campania (68%), Trentino (11%), Veneto (8%), Marche (5%), and Liguria and Puglia (3%). Authors identified levamisole as the most abundant adulterant (81%), followed by single cases of heroin, diltiazem, aminopyrine, hydroxyzine and ketamine, ethylphenidate, and *α*-pyrrolidinovalerophenone (*α*-PVP). Pichini et al. [27] also reported caffeine (19%) as a minor adulterant, associated with phenacetin (16%) and procaine (5%). Those authors also claimed that GC-MS analysis was not able to identify whether the racemate of any of the enantiomers was present. Therefore, they recommended that further work might be needed to identify the enantiomer and/or confirm the racemate. This recommendation is also extended to this work.

A study carried out in Brazil from July 2008 to May 2010 by Magalhães et al. [28] evaluated cocaine purity and the concentration ranges of adulterants and inorganic constituents for 31 street cocaine samples. Authors claimed that cocaine concentrations in samples seized in the Amazonas state ranged from 154 mg/g to 978 mg/g, and these samples did not contain any of the adulterants studied (caffeine, lidocaine, and benzocaine). On the other hand, the cocaine concentrations in the samples seized in the Minas Gerais state ranged from 64 mg/g to 753 mg/g, being caffeine the main adulterant found in 76% of the samples, ranging in concentration from 6 mg/g to 645 mg/g. Lidocaine was found in 67% of the samples, with concentrations ranging from 16 mg/g to 577 mg/g. Benzocaine was found in only one sample, at a concentration of 85 mg/g. Magalhães et al. [28] also claimed that Minas Gerais and Amazonas showed statistically different purity and adulterant composition of seized cocaine, which was attributed to a heavy drug manipulation in Minas Gerais versus a small alteration in Amazonas, a state closer to Colombia.

In other Brazilian reports, Lapachinske et al. [20] analyzed 54 samples seized by the Brazilian Federal Police in the International Airport of São Paulo and mailing services during the year 2011. Authors associated all the samples with international trafficking and were apprehended while leaving the country. The purity of cocaine ranged from 17% to 91%, and cocaine was the only detected active compound in 30% of samples. Among the identified cutting agents, levamisole was the most abundant (56% samples), and relative concentrations ranged from 1% to 23%. Lidocaine, caffeine, phenacetin, and 4-dimethylaminoantipyrine were also identified in those samples in minor concentrations. As a remarkable finding in the study of Lapachinske et al. [20], drugs intended for international trafficking did not present high cocaine purity, and most of the samples were laced with adulterants before leaving Brazil. Conversely, in cocaine seized in the metropolitan area of São Paulo, cocaine purity was consistently around 70% and free of adulterants in 73% of cases, indicating that high purity cocaine enters directly in the form of coca paste from nearby source countries [29].

In the European Union context, Brunt et al. [30] published a work involving an international collaborative effort called the Trans European Drug Information (TEDI) project, which combined data of drug testing systems from of Spain, Switzerland, Belgium, Austria, Portugal, and the Netherlands. Authors reported that between 2011 and 2013, France, Luxembourg, and Austria reported purity of street cocaine between 30% and 43%, lower than the values reported by the Basque Country (Spain) and the Netherlands and Switzerland (roughly 60%).

Finally, in their review, Solimini et al. [31] claimed that pharmacologically active substances such as levamisole, phenacetin, lidocaine, hydroxyzine, and diltiazem have been identified in cocaine over the years. These authors also stated that since cocaine is extracted from natural products, some impurities and minor alkaloids can be present in the final preparation.

### 3.2. Purity and Adulterants Found in Coca Paste Samples

The purity and main adulterants found in samples of coca paste, better known as bazuco, are given in Table 2. In Table 2, different superscript letters in the same column indicate statistically significant differences (*p* < 0.05) in the content of the compound in doses from different neighborhoods around Cartagena de Indias. As in the case of cocaine, all the personal doses of coca paste analyzed were found to be adulterated. In addition to cocaine, caffeine, phenacetin, levamisole, and lidocaine were identified in the samples analyzed, along with other unidentified components. Wheat flour, brick powder, brown sugar, lactose, talc, and mannitol could have been the other additives present and not identified in the analyzed samples [32].

The cocaine concentration in samples from all neighborhoods showed statistically significant differences (*p* < 0.05) in its content, with minimum values of 14% in the San Francisco neighborhood, contrasting with a maximum of 60% in the La Esperanza neighborhood. These data coincide with studies conducted by Sabogal-Carmona and Urrego-Novoa [33]. In their report on the chemical composition of coca paste samples seized in Colombia during the first semester of 2010, the authors reported cocaine concentrations between 20% and 50%.

Caffeine was detected in 100% of the coca paste samples analyzed, and there were statistically significant differences (*p* < 0.05) in its content in samples from all neighborhoods of the city. Its concentration ranged from 2% in samples from the Candelaria neighborhood to a maximum of 20% in samples from the La Esperanza neighborhood.

Regarding phenacetin, this adulterant was present in 70% of coca paste samples from the analyzed neighborhoods; it was not identified in samples from La Quinta, Líbano, and La María. Its maximum content was found in personal doses from the San Francisco neighborhood, while the minimum was identified in the Zapatero neighborhood, where it barely reached 1%.

On the other hand, levamisole was only detected in 60% of the personal doses of coca paste analyzed, with minimum concentrations of 1% in the San Francisco and Zapatero neighborhoods, up to a maximum of 18% in the La María neighborhood.

A new pharmaceutical found in coca paste samples, which was not detected in cocaine, was lidocaine, found in samples from half of the neighborhoods analyzed, with significant differences (*p* < 0.05) in the samples where it was identified. Lidocaine, also known as lignocaine, is a local anesthetic of the amino amide-type. It is an antiarrhythmic drug that works by blocking sodium pathways and thereby slowing the rate of heart contractions. It is also used to treat ventricular tachycardia. This adulterant can cause brain effects such as confusion, vision changes, numbness, tingling, and vomiting. It can also cause low blood pressure and irregular heart rate [20].

Barbera et al. [34] reported five (5) cases of drug overdose in Italy in which lidocaine, used as an adulterant substance, has had a role in the pathogenic mechanism of death. Considering the pharmacological mechanism, blood concentration, and route of administration, the authors hypothesized that lidocaine could act with a synergistic (or at least additive) effect with the illicit drug on the central nervous system and the cardiovascular system.

## 4. Conclusions

This is the first attempt to characterize the composition of street cocaine and coca paste samples from Cartagena de Indias. However, the finds are preliminary given that they only derived from donated samples.

All the personal doses donated from different strategic neighborhoods of the city of Cartagena de Indias (Colombia), both cocaine hydrochloride (cocaine or perico) and coca base paste (bazuco), were adulterated and did not show a high purity of cocaine. Most of the samples were mixed with active chemicals, which can modify the signs and symptoms of cocaine intoxication. Caffeine, phenacetin, and levamisole were the main adulterants identified in cocaine and coca paste; although for the latter, lidocaine could also be detected. According to other reports [32], the rest of the unidentified components could have been additives used to increase yields, such as wheat flour, brick powder, brown sugar, lactose, talc, and mannitol. The results are consistent with other research in terms of the widespread use of caffeine as an adulterant, but they also follow the growing trend of using levamisole and phenacetin as adulterants. These adulterants can not only be used to dilute drugs and increase yield but act as enhancers, which are also pharmacologically active compounds.

The wide range of cocaine content in samples sold in the illicit market distributed in key neighborhoods of the city of Cartagena de Indias could cause undesirable effects on cocaine users who do not know the exact intended dose for consumption. This study provides new relevant data on personal doses adulterated with compounds that could represent a public health problem and should be addressed in drug use prevention and law enforcement strategies in the country. This study could thus contribute to improving public health responses to the addiction to these types of drugs that are widely used and relatively easy to access in the city of Cartagena de Indias and Colombia in general.

Drug use is higher in urban than in rural areas in both developed and developing countries. Colombia is no exception, and the massive displacement of people from the countryside to the cities partly explains the overall increase in drug use in the country and indicates that it could get worse. There is also an evident and constant presence of national and international travelers in the Tourist and Cultural District of Cartagena de Indias during any time of the year, even in nonholiday seasons. Most of the tourists arriving in the city come from the United States (31%), and it is no secret that one of the reasons for the international visit is the ease in obtaining illicit substances such as cocaine and coca paste, along with to the low cost compared to the international standard. Information on the actual composition of illegal drugs can be useful to inform health specialists and consumers who have limited knowledge about the composition of drugs sold on the illicit market. Therefore, this study, in addition to making this information useful to academic, public health, and national security agencies (since the identification of adulterants provides relevant information for the investigation of trafficking routes and drug profiling) and other government agencies, is also accessible to tourists entering the city, so that they are aware of what they are actually consuming and the risks to which they are exposed.

## Figures and Tables

**Table 1:** Purity and adulterants found in cocaine samples (perico).

Component	Getsemaní	Zona Centro	Sector Bomba del Amparo	La Quinta	Líbano
Cocaine (%)	63.5 ± 0.6^a^	65.7 ± 0.7^b^	67.5 ± 1.0^c^	30.5 ± 0.8^d^	8.0 ± 0.0^e^
Caffeine (%)	10.1 ± 0.0^a^	Not detected	6.0 ± 0.0^b^	41.64 ± 0.9^c^	40.5 ± 0.6^c^
Phenacetin (%)	9.8 ± 0.0^a^	17.2 ± 0.0^b^	8.0 ± 0.0^c^	Not detected	Not detected
Levamisole (%)	15.0 ± 0.0^a^	2.0 ± 0.0^b^	9.0 ± 0.0^c^	Not detected	Not detected

**Table 2:** Purity and adulterants found in coca paste samples (bazuco).

Neighborhood	Component (%)				
	Cocaine	Caffeine	Phenacetin	Levamisole	Lidocaine
Getsemaní	30.0 ± 0.1^a^	6.0 ± 0.0^a^	4.0 ± 0.0^a^	Not detected	Not detected
Centro de la ciudad	45.0 ± 0.6^b^	15.0 ± 0.0^b^	9.0 ± 0.0^b^	Not detected	Not detected
Sector Bomba del Amparo	23.0 ± 0.2^c^	12.0 ± 0.0^c^	20.0 ± 0.0^c^	Not detected	Not detected
La Quinta	34.0 ± 0.7^d^	15.0 ± 0.1^b^	Not detected	Not detected	Not detected
Líbano	15.0 ± 0.0^e^	4.0 ± 0.0^d^	Not detected	2.0 ± 0.0^a^	Not detected
La Esperanza	60.0 ± 1.0^f^	20.0 ± 0.8^e^	4.0 ± 0.0^a^	5.0 ± 0.0^b^	2.0 ± 0.0^a^
La Candelaria	36.0 ± 0.5^g^	2.0 ± 0.0^f^	17.0 ± 0.0^d^	7.0 ± 0.0^c^	18.0 ± 0.0^b^
La María	38.0 ± 0.4^h^	4.0 ± 0.0^d^	ND	18.0 ± 0.0^d^	40.0 ± 0.9^c^
San Francisco	14.0 ± 0.1^i^	11.0 ± 0.0^g^	28.0 ± 0.1^e^	1.0 ± 0.0^e^	5.0 ± 0.0^d^
Zapatero	20.0 ± 0.4^j^	15.0 ± 0.1^b^	1.0 ± 0.0^f^	1.0 ± 0.0^e^	48.0 ± 1.0^e^

## Data Availability

The data used to support the findings of this study are available from the corresponding author upon request.
